# Prognostic Characteristics and Immune Effects of N^6^-Methyladenosine and 5-Methylcytosine-Related Regulatory Factors in Clear Cell Renal Cell Carcinoma

**DOI:** 10.3389/fgene.2022.864383

**Published:** 2022-04-27

**Authors:** Lei Li, Zijia Tao, Yiqiao Zhao, Mingyang Li, Jianyi Zheng, Zeyu Li, Xiaonan Chen

**Affiliations:** Department of Urology, Shengjing Hospital of China Medical University, Shenyang, China

**Keywords:** renal clear cell cancer, N6-methyladenose, prognosis, The Cancer Genome Atlas, 5-methylcytosine, tumor microenvironment

## Abstract

In recent years, methylation modification regulators have been found to have essential roles in various tumor mechanisms. However, the relationships between N^6^-methyladenosine (m6A) and 5-methylcytosine (m5C) regulators and clear cell renal cell carcinoma (ccRCC) remain unknown. This study investigated these relationships using the data from The Cancer Genome Atlas database. We calculated risk scores using a Lasso regression analysis and divided the patient samples into two risk groups (tumor vs. normal tissues). Furthermore, we used univariate and multivariate Cox analyses to determine independent prognostic indicators and explore correlations between the regulatory factors and immune infiltrating cell characteristics. Finally, quantitative reverse transcriptase–polymerase chain reaction (PCR) and The Human Protein Atlas were used to verify signature-related gene expression in clinical samples. We identified expression differences in 35 regulatory factors between the tumor and normal tissue groups. Next, we constructed a five-gene risk score signature (NOP2 nucleolar protein [*NOP2*], methyltransferase 14, N6-adenosine-methyltransferase subunit [*METTL14*], NOP2/Sun RNA methyltransferase 5 [*NSUN5*], heterogeneous nuclear ribonucleoprotein A2/B1 [*HNRNPA2B1*], and zinc finger CCCH-type containing 13 [*ZC3H13*]) using the screening criteria (*p* < 0.01), and then divided the cases into high- and low-risk groups based on their median risk score. We also screened for independent prognostic factors related to age, tumor grade, and risk score. Furthermore, we constructed a Norman diagram prognostic model by combining two clinicopathological characteristics, which demonstrated good prediction efficiency with prognostic markers. Then, we used a single-sample gene set enrichment analysis and the cell-type identification by estimating relative subsets of RNA transcripts (CIBERSORT) method to evaluate the tumor microenvironment of the regulatory factor prognostic characteristics. Moreover, we evaluated five risk subgroups with different genetic signatures for personalized prognoses. Finally, we analyzed the immunotherapy and immune infiltration response and demonstrated that the high-risk group was more sensitive to immunotherapy than the low-risk group. The PCR results showed that *NSUN5* and *HNRNPA2B1* expression was higher in tumor tissues than in normal tissues. In conclusion, we identified five m6A and m5C regulatory factors that might be promising biomarkers for future research.

## Introduction

Renal cell carcinoma (RCC) is the most common renal malignancy, resulting in more than 14,000 deaths annually in the United States (Siegel et al., 2018). Clear cell renal cell carcinoma (ccRCC) is the most common subtype (∼70% of cases) with the worst degree of malignancy and prognosis (Jonasch et al., 2021). There has been considerable progress regarding ccRCC treatment. However, overall survival (OS) and relapse-free survival still require improvement (Greef and Eisen, 2016). Therefore, identifying new targets and prognostic biomarkers for ccRCC treatment is crucial.

N-Methyladenosine (m6A) and 5-methylcytosine (m5C) RNA modifications are newly discovered gene expression regulation mechanisms (Chen et al., 2019). These modifications affect the fate of modified RNA and play critical roles in biological processes such as tumor development (Huang et al., 2020). m6A modifications are the most abundant type of RNA modification, occurring in messenger RNA (mRNA), microRNA, and long noncoding RNAs. Furthermore, studies have demonstrated that m6A RNA modifications affect RNA processing, translation, and metabolism. Methyltransferases (writers), demethylases (erasers), and binding-protein (readers) primarily mediate the effects of m6A. The “writers” are responsible for RNA methylation and include methyltransferase-like (METTL) 33, METTL14, KIAA1429, Wilms’ tumor 1-associating protein (WTAP), RNA binding motif protein 15 (RBM15), and zinc finger CCCH-type containing 13 (ZC3H13). The “erasers” specifically target m6A RNA and mainly include AlkB homolog 5, RNA demethylase (ALKBH5), and FTO alpha-ketoglutarate-dependent dioxygenase (FTO). The “readers” connect m6A sites and play a role in special regulatory RNA modifications, including YTH domain-containing (YTHDC) 1, YTHDC2, YTH domain-containing family protein (YTHDF) 1, YTHDF2, insulin-like growth factor binding protein (IGFBP) 1, IGFBP2, IGF2BP3, RNA binding motif protein X-Linked (RBMX), and heterogeneous nuclear ribonucleoprotein C (HNRNPC). m6A downregulation leads to reduced proliferation, self-renewal, survival, and differentiation. Furthermore, m6A methylation regulates all aspects of cellular RNA metabolism, including abundance, alternative splicing, stability, nuclear output, decay, and transformation (Chen et al., 2019; He et al., 2019; Lan et al., 2019).

m5C modifications are another prevalent RNA modification type occurring in mRNA, transfer RNA, ribosomal RNA, and some noncoding RNAs (Squires et al., 2012; Bohnsack et al., 2019; Trixl and Lusser, 2019). Studies have demonstrated that m5C is involved in gene expression related to RNA output, translation, and stabilization processes (Bohnsack et al., 2019; Huang et al., 2019). Writers, readers, and erasers also mediate the effects of m5C. NOP2 nucleolar protein (NSUN) 1–7, DNA methyltransferase (DNMT) 1, 2, 3A, and 3B are writers, regulating the process of RNA methylation modifications. Tet methylcytosine dioxygenase (TET) 2, an eraser, has m5C demethylation activity, removing the m5C modification, and Aly/REF export factor (ALYREF), a reader, recognizes and binds the m5C site on target mRNAs (Huang et al., 2019; Nombela et al., 2021).

Increasing evidence suggests that m6A and m5C regulators have essential roles in tumorigenesis and tumor progression (Chen et al., 2019; Chellamuthu and Gray, 2020). For example, tumorigenesis and proliferation, differentiation, invasion, and migration are related to methylation modifications (Lin et al., 2016; Ma et al., 2017; Liu et al., 2018a). Furthermore, m6A and m5C regulators have been reported as prognostic biomarkers. For instance, in hepatocellular carcinoma, METTL3 is associated with a poor prognosis and inhibits the suppressor of cytokine signaling 2 (i.e., SOCS2) expression through the miR-145/m 6 A/YTHDF2-dependent axis (Yang et al., 2017; Chen et al., 2018). In addition, METTL14 promotes cancer progression by regulating MYB proto-oncogene–transcription factor (i.e., MYB)/MYC proto-oncogene–bHLH transcription factor (i.e., MYC) in acute myeloid leukemia (Weng et al., 2018). m5C research has not become mainstream. However, studies have confirmed increased *NSUN2* expression in breast cancer (Frye and Watt, 2006), and *NOP2* is a non–small cell lung cancer prognostic biomarker (Sato et al., 1999). Furthermore, *NSUN5* is highly expressed in rectal cancer and promotes cancer progression through cell cycle regulation (Jiang et al., 2020).

RNA modifications do not drive tumor progression. However, abnormal expression of modification regulators can lead to changes in the biological behavior of tumors (Nombela et al., 2021). For example, *METTL3* is upregulated in breast cancer, which increases hepatitis B X-interacting protein (i.e., *HBXIP*) mRNA methylation and stability, inducing tumor cell proliferation and survival by inhibiting the tumor suppressor, let −7 g (Cai et al., 2018). METTL3 also regulates integrin subunit beta 1 (ITGB1) expression, thereby affecting the binding of ITGB1 to collagen I. This disruption affects tumor cell migration and promotes bone metastasis in prostate cancer (Li et al., 2020). In cervical cancer, FTO activates the β-catenin pathway, increasing ERCC excision repair 1–endonuclease noncatalytic subunit (i.e., *ERCC1*) expression, which is associated with worse prognosis (Zhou et al., 2018; Zou et al., 2019). In addition, FTO is overexpressed in lung cancer, promoting cell proliferation and invasion and inhibiting apoptosis by regulating myeloid zinc finger 1 (i.e., *MZF1*) expression, resulting in a poor prognosis (Liu et al., 2018b). This evidence demonstrates that RNA methylation significantly influences the biological behavior of tumors.

Many studies have explored the regulatory mechanisms among m6A- and m5C-related regulatory factors and various tumors. However, relationships between the clinicopathological characteristics of ccRCC and combined m6A–m5C regulatory factors remain unclear. Therefore, this study combined the gene signatures of m6A with m5C to explore these correlations. We downloaded kidney renal clear cell carcinoma (KIRC) transcriptome and clinical data from The Cancer Genome Atlas (TCGA) to analyze the differentially expressed regulatory factors in ccRCC. Next, we constructed a prognostic risk model using Lasso regression and Cox analyses. Finally, we screened five prognostic regulatory factors as a model signature and combined the independent prognostic factors to construct a nomogram diagram.

Immune cell infiltration plays a decisive role in tumorigenesis and development. Furthermore, cancer cells shape their microenvironment by secreting various cytokines, chemokines, and other factors, leading to the reprogramming of surrounding cells. Therefore, they play a decisive role in tumor survival and progression (Hinshaw and Shevde, 2019). This study also aimed to identify the potential characteristics of m6A- and m5C-related regulatory factors to improve prognostic ccRCC evaluations.

Overall, we combined m6A and m5C to explore the influence of regulatory factors on ccRCC prognoses to provide new ccRCC biomarkers and construct a reliable prognostic model suitable for use in the clinic.

## Methods

### Data Collection and Processing

We downloaded the ccRCC transcriptome (HTseq-FPKM) and clinical data from the TCGA-KIRC database (https://portal.gdc.cancer.gov/). We included 611 TCGA samples; 539 were tumor tissue (ccRCC) samples, and 72 were normal tissues samples. We removed all samples with missing data.

### Expression Differences in N^6^-methyladenosine and 5-methylcytosine-Related Regulatory Factors

We included 23 m6A and 12 m5C regulatory factors based on the literature (Bohnsack et al., 2019; He et al., 2019; Zhang et al., 2021) (m6A: KIAA1429, WTAP, RBM15, RBM15B, METTL16, METTL3, METTL14, ZC3H13, ALKBH5, FTO, FMRP translational regulator 1 [FMR1], heterogeneous nuclear ribonucleoprotein A2/B1 [HNRNPA2B1], HNRNPC, IGFBP1, IGFBP2, IGFBP3, leucine-rich pentatricopeptide repeat-containing [LRPPRC], RBMX, YTHDC1, YTHDC2, YTHDF1, YTHDF2, and YTHDF3; m5C: TET1, TET2, TET3, NSUN2, NSUN3, NSUN4, NSUN5, NSUN6, NOP2, ALYREF, tRNA aspartic acid methyltransferase 1 [TRDMT1], and YBX1). We compared the gene expressions of these regulatory factors between the tumor and normal tissue groups. A *p*-value of <0.05 and an absolute log2 fold change value ≥ 1 were considered statistically significant.

### Prognostic Characteristics of N^6^-methyladenosine- and 5-methylcytosine-Related Regulatory Factors

We randomly divided the 525 ccRCC samples into training (*n* = 263) and validation (*n* = 262) groups using a 1:1 ratio and screened the prognostic adjustment factors. We calculated the risk score as follows:

$$\text{Riskscore}=\left(Exp_{gene1}\times Coef_{gene1}\right)+\left(Exp_{gene2}\times Coef_{gene2}\right)+\cdots +\left(Exp_{gene\left(n\right)}\times Coef_{gene\left(n\right)}\right),$$

where Exp: individual gene expression; Coef: correlation coefficient.

The samples were classified into high- and low-risk groups based on the median risk score. Next, we used univariate and multivariate Cox regression analyses to determine which pathological features were independent prognostic risk factors. Finally, we prepared receiver operating characteristic (ROC) curves. This process was performed in the training and validation groups.

### Nomogram Prognostic Model Construction

We selected clinical indicators with a *p*-value of ≤0.001 in the multivariate Cox regression analysis and the risk score to construct the prognostic nomogram model. The calibration curve and C-index were used to predict the model’s performance. We also created 1-, 3-, and 5-year ROC curves to verify the model’s predictive ability and performed a Kaplan–Meier analysis to evaluate the OS of the low- and high-risk groups (statistical significance was set at *p* < 0.05).

### Cell Infiltration in the Tumor Microenvironment

We used the single-sample gene set enrichment analysis (ssGSEA) algorithm to obtain the relative abundance of TME-infiltrated cells per sample. Next, we used the CIBERSORT algorithm to analyze differences in immune cell infiltration between the high- and low-risk groups. The CIBERSORT software deconvolves the matrix of immune cell subtypes according to linear support vector regression rules (Lee et al., 2013). We downloaded the ccRCC immune scores from the MD Anderson database (http://bioninformatics.mdanderson.org/estimate/) to assess correlations between regulatory factors and immune and matrix scores.

### Cell Culture, Quantitative Real-Time Polymerase Chain Reaction, and Signature Gene Expression Analyses

We purchased human ccRCC (769-P) and immortalized proximal tubule epithelial (HK2) cell lines from the Cell Bank of the Chinese Academy of Sciences. 769-P and HK2 cells were cultured in Roswell Park Memorial Institute (i.e., RPMI)-1,640 medium (KeyGEN Biotech, Inc., Nanjing, China) and Dulbecco’s Modified Eagle Medium (i.e., DMEM) (KeyGEN Biotech, Inc.,), respectively, containing 10% fetal bovine serum (Biological Industries, Shanghai, China) at 37°C and 5% carbon dioxide.

We verified the expression levels of the prognostic genes by qRT-PCR analysis. Total RNA was extracted using Trizol reagent (TaKaRa Bio Inc. Shiga, Japan), and complementary DNA was synthesized using PrimeScript RT reagent Kit (TaKaRa, Shiga, Japan). qRT-PCR was performed on 7,500 Real-Time PCR Systems (Applied Biosystems; Thermo Fisher Scientific, Waltham, MA, United States) using SYBR GreenER Supermix (TaKaRa, Shiga, Japan). The PCR conditions comprised an initial melting step at 95°C for 1 min, followed by 35 cycles of 95°C for 90 s, 60°C for 30 s, 72°C for 30 s, and then 72°C for 10 min. We used the 2^–ΔΔCt^ method to analyze the relative expression of the prognostic genes based on the normalized relative expression of the β-actin gene. The primers were as follows: NSUN5: Forward, TGC​CTC​GAT​TTG​TGC​GTG​TG, Reverse, GAC​AGC​TGG​CCC​TGT​CCT; GAPDH: Forward, TGA​CTT​CAA​CAG​CGA​CAC​CCA, Reverse, CAC​CC-TGT​TGC​TGT​AGC​CAA​A; ZC3H13: Forward, TGG​TGC​TGG​AGA​AGG​ATA​CGA, Reverse, CTA​TCA​CAT​CTA​AGG​GAT​CTG​GCA; HNRNPA2B1: Forward, GCT​TTG​GGG​ATT​CAC​GTG​GT, Reverse, CCA​CTG​CCA​TAT​CCA​TCA​GAT​CC. We used The Human Protein Atlas database to analyze NSUN5 and HNRNPA2B1 protein expression in clinical specimens.

### Statistical Analyses

All statistical analyses were performed using R (version 3.6.1; R Foundation for Statistical Computing, Vienna, Austria). We created box plots to visualize differential gene expression using the “reshape2” and “ggpubr” packages. We screened the prognostic adjustment factors using a Lasso regression analysis and the “glmnet” package and generated ROC curves using the “survivalROC” package. The survival curve was obtained using the “survminer” package, and we used the “rms” package to calculate the C-index and generate calibration curves. All *p*-values of <0.05 were considered statistically significant. We indicated the various levels of statistical significance as follows: *p < 0.05, **p < 0.01, and ***p < 0.001.

## Results

### Expression Patterns of the N^6^-methyladenosine and 5-methylcytosine Regulatory Factors

**FIGURE 1:**
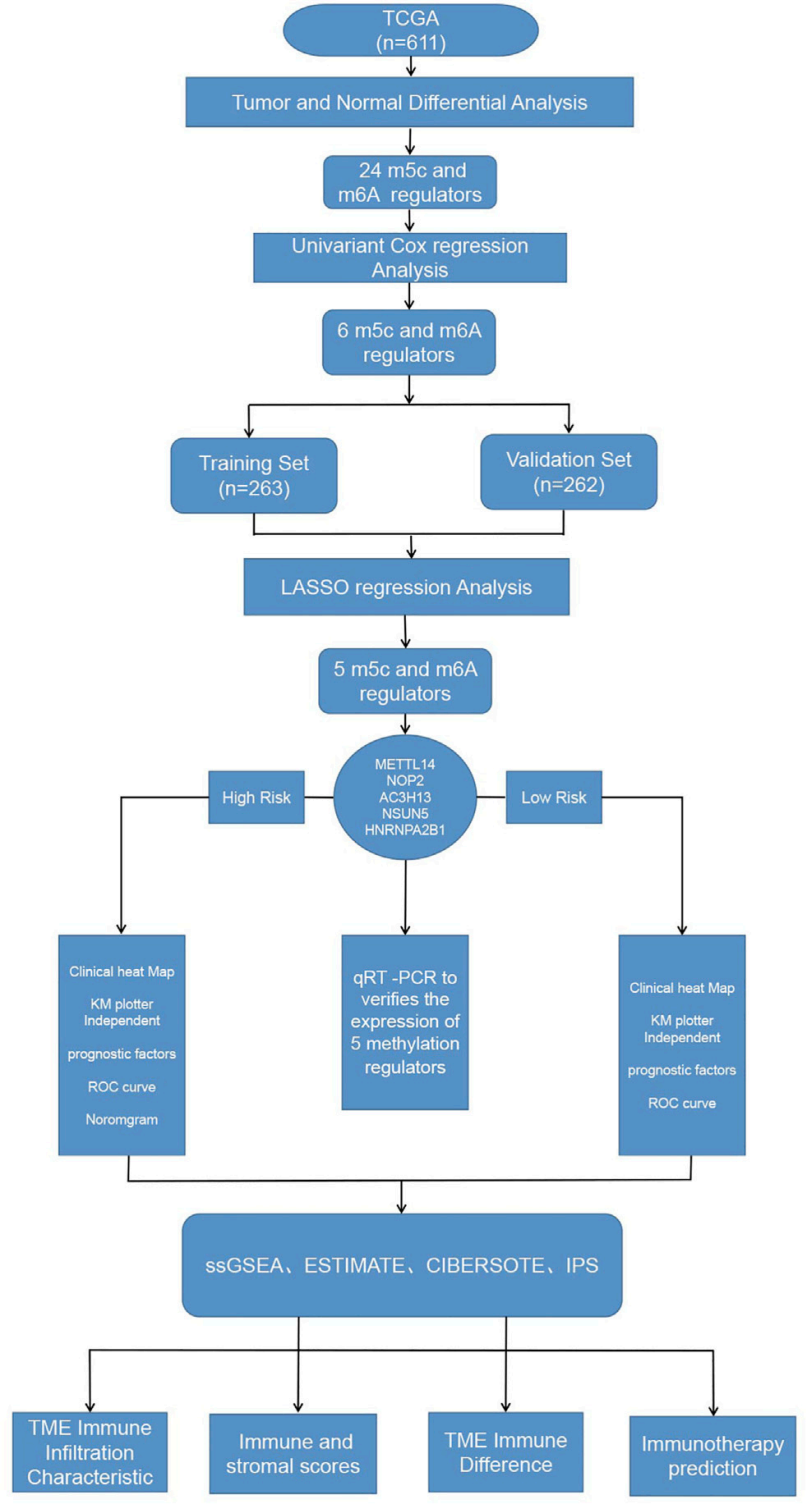
Workflow of this study.

**FIGURE 2:**
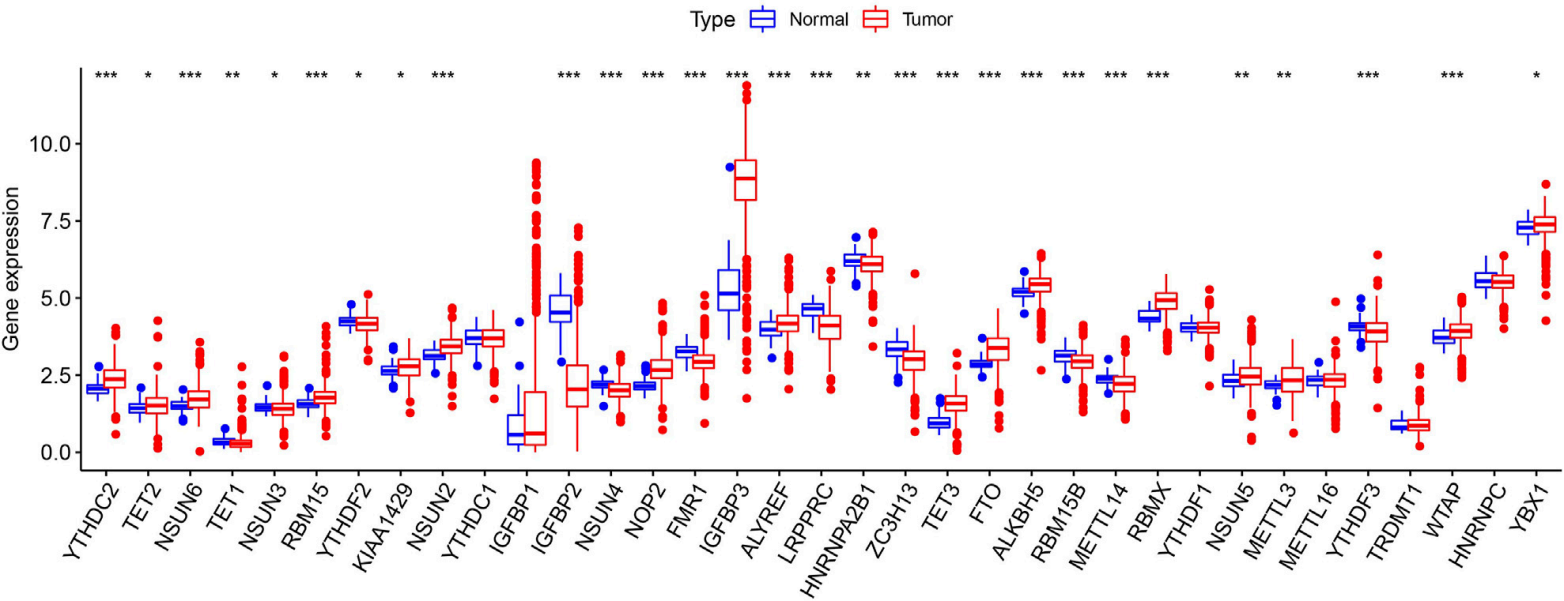
Expression pattern of m6A and m5C regulators in TCGA ccRCC cohort. Difference expression of 35 m6A and m5C methylation regulators. *p < 0.05, **p < 0.01, and ***p < 0.001.

Figure 1presents a workflow chart, and Table 1 lists the combined (m6A and m5C) regulatory factors. We identified 24 genes that differed between the tumor and normal tissue groups (*p* < 0.01; Figure 2). The expressions of *NSUN4*, *FMR1*, *LRPPRC*, *HNRNPA2B1*, *ZC3H13*, *RBM15B*, *METTL14*, *YTHDF3*, and *IGFBP2* were significantly higher in the normal tissue samples than in the tumor tissue samples. The expressions of *YTHDC2*, *NSUN6*, *RBM15*, *NSUN2*, *NOP2*, *IGFBP3*, *ALYREF*, *TET3*, *FTO*, *ALKBH5*, *RBMX*, *NSUN5*, *METTL3*, and *WTAP* were significantly higher in the tumor tissue samples than in the normal tissue samples. *TET1* expression did not differ among the groups (Supplementary Table S1).

**TABLE 1:** Classification of m6A and m5C combined regulatory factors.

Name	Regulators	Type
m6A	METTL3	writer
	METTL14	writer
	METTL16	writer
	YTHDF1	reader
	YTHDF2	reader
	YTHDF3	reader
	YTHDC1	reader
	YTHDC2	reader
	RBM15	writer
	RBM15 B	writer
	RBMX	reader
	IGFBP1	reader
	IGFBP2	reader
	IGFBP3	reader
	KIAA1429	writer
	FMR1	reader
	LRPPRC	reader
	HNRNPA2B1	reader
	HNRNPC	reader
	ZC3H13	writer
	FTO	eraser
	ALKBH5	eraser
	WTAP	writer
m5C	TET1	eraser
	TET2	eraser
	TET3	writer
	NOP2	writer
	NSUN2	writer
	NSUN3	writer
	NSUN4	writer
	NSUN5	writer
	NSUN6	writer
	ALYREF	reader
	TRDMT1	writer
	YBX1	reader

### Kidney Renal Clear Cell Carcinoma Prognostic Model Combined With Regulatory Factors

**FIGURE 3:**
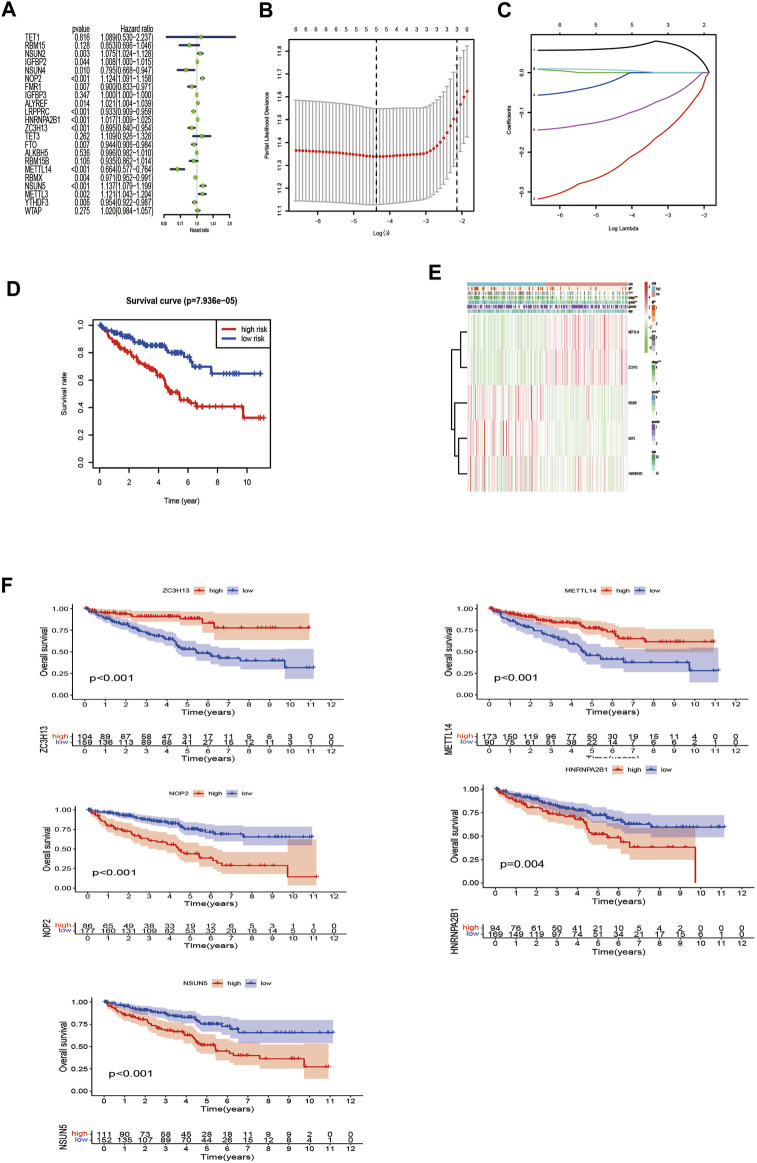
Construction of the risk signature according to the m6A and m5C RNA methylation regulators. **(A)** Forest plot of the univariate Cox regression analysis for the 22 regulators. Identification of six significant regulators (*p < 0.05, **p < 0.01, and ***p < 0.001). **(B,C)** LASSO coefficient profiles of the six regulators. Cross-validation for tuning parameter selection in the LASSO model. **(D)** The K–M analysis showed that patients in the low-risk group presented better OS than those in the high-risk group. This analysis was based on the survival information of samples in the training set. The red line represents the high-risk cluster, whereas the blue line indicates the low-risk cluster. **(E)** The training set of the heat map of the relationship between the gene expression of the corresponding five regulatory factors and clinical features. *p < 0.05, **p < 0.01, and ***p < 0.001. **(F)** Kaplan–Meier survival curves for OS of five regulatory factors.

We further screened the methylation regulatory factors to explore their prognostic value. We selected 22 regulatory factors as the analysis object (Figure 3A). Univariate Cox regression analysis identified six regulatory factors related to OS (Supplementary Table S2), and the Lasso regression analysis identified five relevant prognostic factors, namely, *NOP2*, *METTL14*, *NSUN5*, *HNRNPA2B1*, and *ZC3H13* (Figures 3B,C; Supplementary Table S3). *NSUN5*, *NOP2*, and *HNRNPA2B1* were associated with risk (hazard ratio [HR] >1), and *METTL14* and *ZC3H13* were associated with a protective effect (HR <1). We used these five factors to generate the prognostic risk model.

Next, we compared the survival rates of the high- and low-risk groups based on the median risk. The survival rate of the low-risk group was distinctly better than that of the high-risk group (Figure 3D). Furthermore, the heat map illustrated relationships among the gene expressions of the five prognostic factors from the training set and various clinical traits and risk scores. The risk score correlated with tumor (T), metastasis (M), stage, and grade. In addition, the highly expressed genes *NOP2*, *HNRNPA2B1*, and *NSUN5* were highly expressed in the high-risk group, and the low-expressed genes *METTL14* and *ZC3H13* were highly expressed in the low-risk group (Figure 3E). High expression of *NOP2*, *HNRNPA2B1*, and *NSUN5* correlated with a poor prognosis, and high *METTL14* and *ZC3H13* expression correlated with a good prognosis (Figure 3F). These results support the results of our predicted risk genes and protective genes.

### Validation of the Risk Model Related to Five Regulators

**FIGURE 4:**
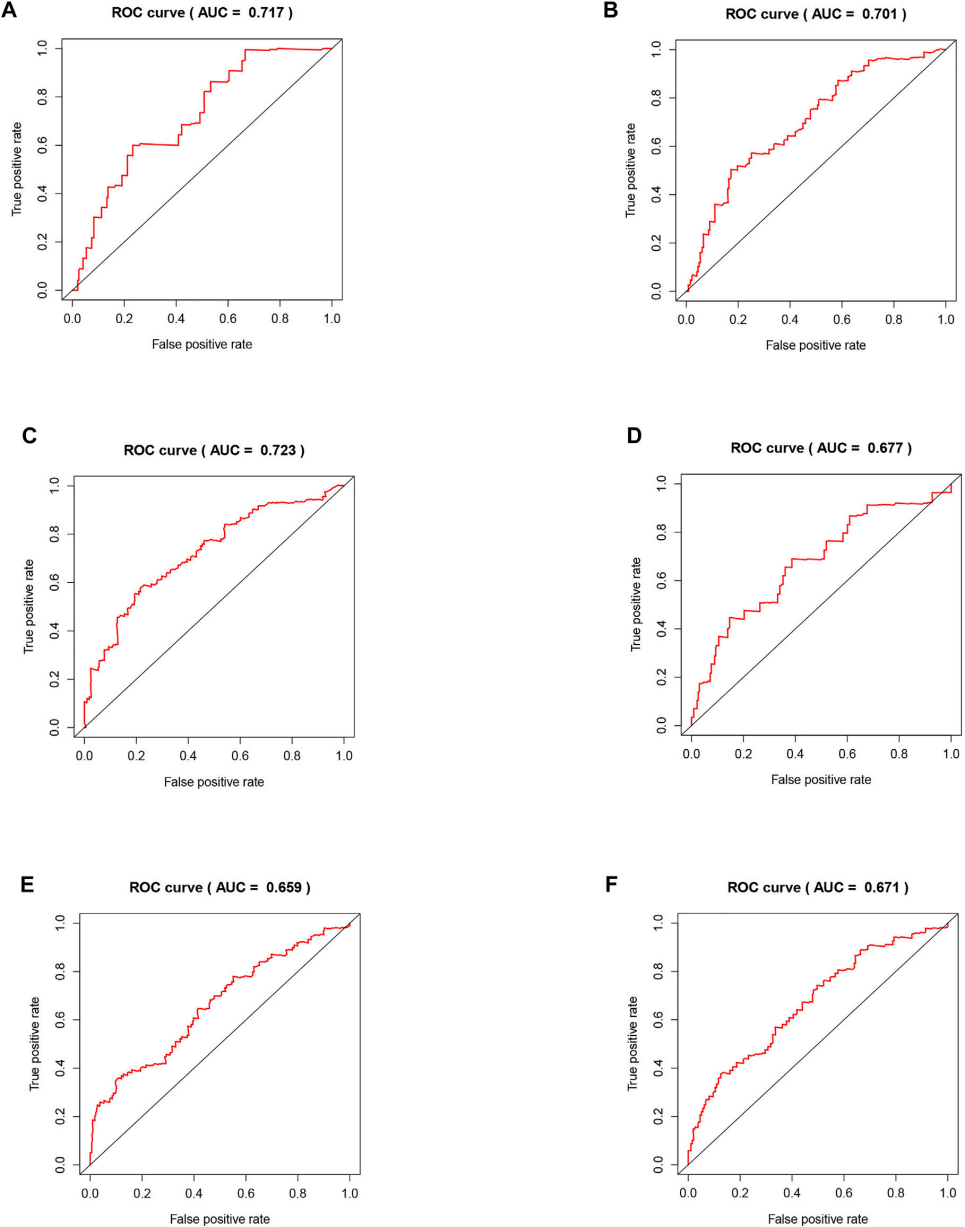
Validation of the risk model related to five regulators. **(A–C)** The training set of the ROC curve for evaluating the prediction efficiency of the prognostic signature. **(D–F)** The validation set of the ROC curve for evaluating the prediction efficiency of the prognostic signature.

**FIGURE 5:**
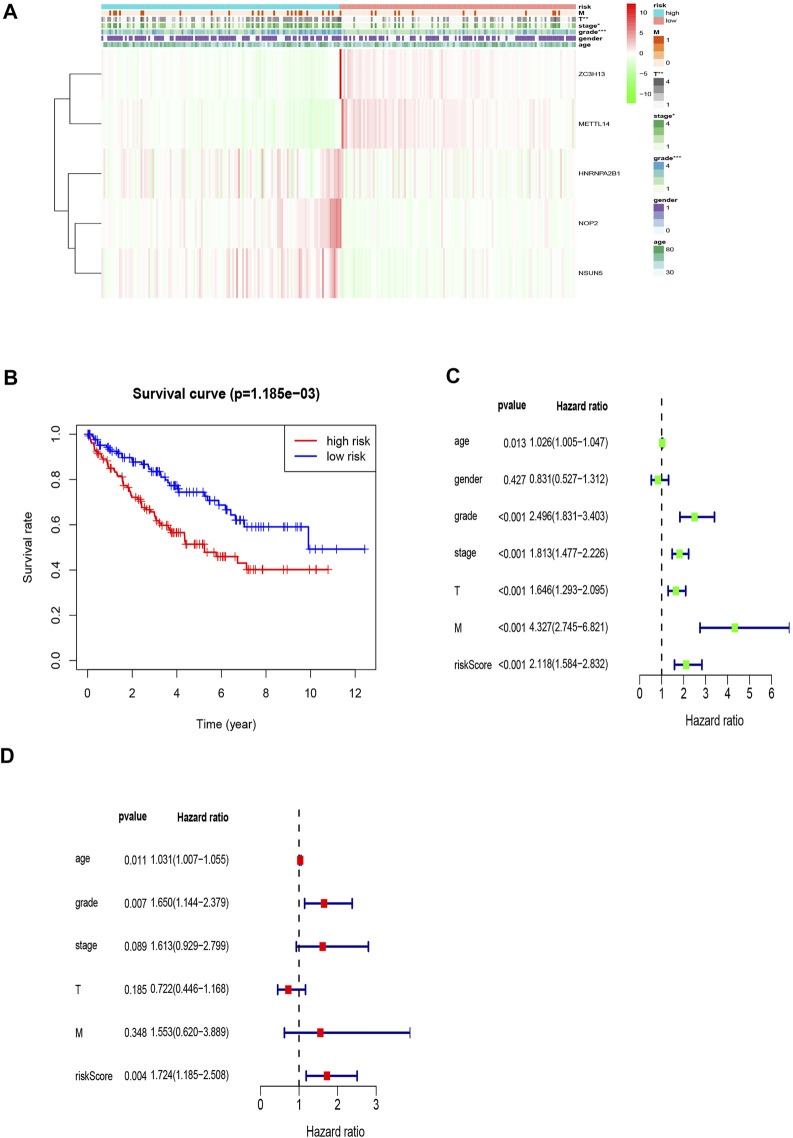
Prognostic signature of the five related regulators in the validation set and the training set of independent prognostic factors for KIRC OS. **(A)** The validation set of the heat map of the relationship between the gene expression of the corresponding five regulatory factors and clinical features. **(B)** Kaplan–Meier survival curves for OS in the two groups of low and high risk. This analysis was based on the survival information of samples in the validation set. **(C,D)** Forrest plot of the independent prognostic factors in KIRC.

The areas under the curve (AUCs) for the 1-, 3-, and 5-year ROC curves were 0.717, 0.701, and 0.723, respectively, using the training set data and the prognostic risk model (Figures 4A–C). We performed the same analysis using the validation set data, finding that the AUCs for the 1-, 3-, and 5-year ROC curves were 0.677, 0.671, and 0.659, respectively (Figures 4D–F). Furthermore, the expression patterns of prognostic regulatory factors in the high- and low-risk groups were almost the same as those in the training set (Figure 5A), and the Kaplan–Meier analysis of the validation set was consistent with the training set results. Patient survival was better in the low-risk group than in the high-risk group (Figure 5B), indicating that these five regulatory factors positively affect the KIRC prognosis prediction. Next, the univariate analysis identified that prognosis was related to the age, grade, stage, T, M, and risk score. The multivariate analysis showed that the age, grade, and risk score were related to the OS (*p* < 0.05). Therefore, age, grade, and risk score are independent prognostic factors for KIRC OS (Figures 5C,D).

### Norman Graph Model Construction and Verification

**FIGURE 6:**
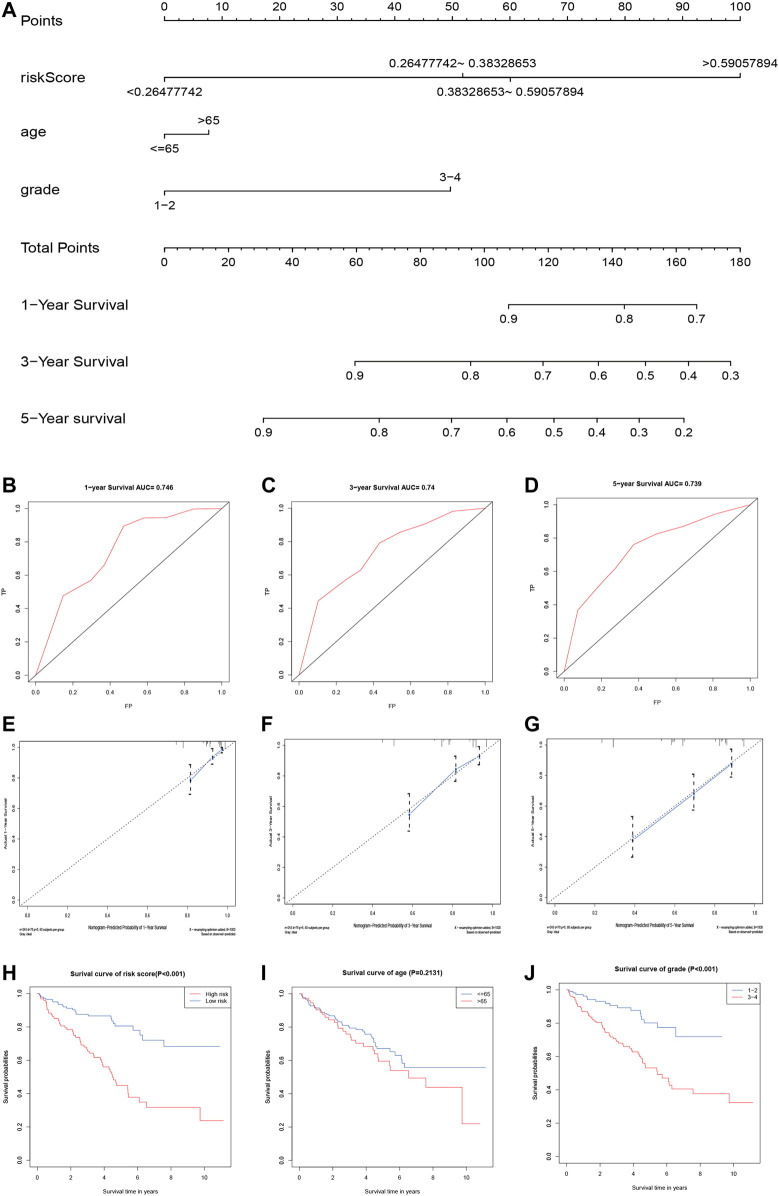
Validation of the prognostic signature of the five related regulators. **(A)** The nomogram of the risk model for predicting the OS probability of ccRCC patients. The whole points projected on the bottom scales indicate the likelihood of 1-, 3-, and 5-year OS. **(B–D)** AUC of the ROC analysis showed the predicted efficacy of the risk model in the training set. **(E–G)** The calibration plot for the nomogram predicting 1-, 3-, and 5-year OS. The *y*-axis indicates the actual survival, as measured by the K–M analysis, while the *x*-axis shows the nomogram-predicted survival. **(H–J)** Kaplan–Meier survival curves stratified according to clinicopathological and risk scores.

We used the independent prognostic factors (i.e., age, grade, and risk score) to construct a Norman diagram model (Figure 6A). Next, we constructed a nomogram based on two independent prognostic factors, and then predicted the 1-, 3-, and 5-year OS using the Norman diagram. The C-index was 0.737, and the calibration curve was more consistent with the observed results. The AUCs for 1-, 3-, and 5-year OS ROC curves were 0.746, 0.740, and 0.739, respectively (Figures 6B–D). The calibration graphs displaying the curves illustrate that the nomogram model has a better predictive ability and accuracy (Figures 6E–G). The grade, age, and risk score survival curves indicate that the survival rate of patients in the early stage was much better than those in the late stage (*p* < 0.001). Furthermore, OS was better in the low-risk group than in the high-risk group (*p* < 0.001; Figures 6H–J).

### Effects of N^6^-methyladenosine Regulatory Factor Modifications on Immune Cell Infiltration

**FIGURE 7:**
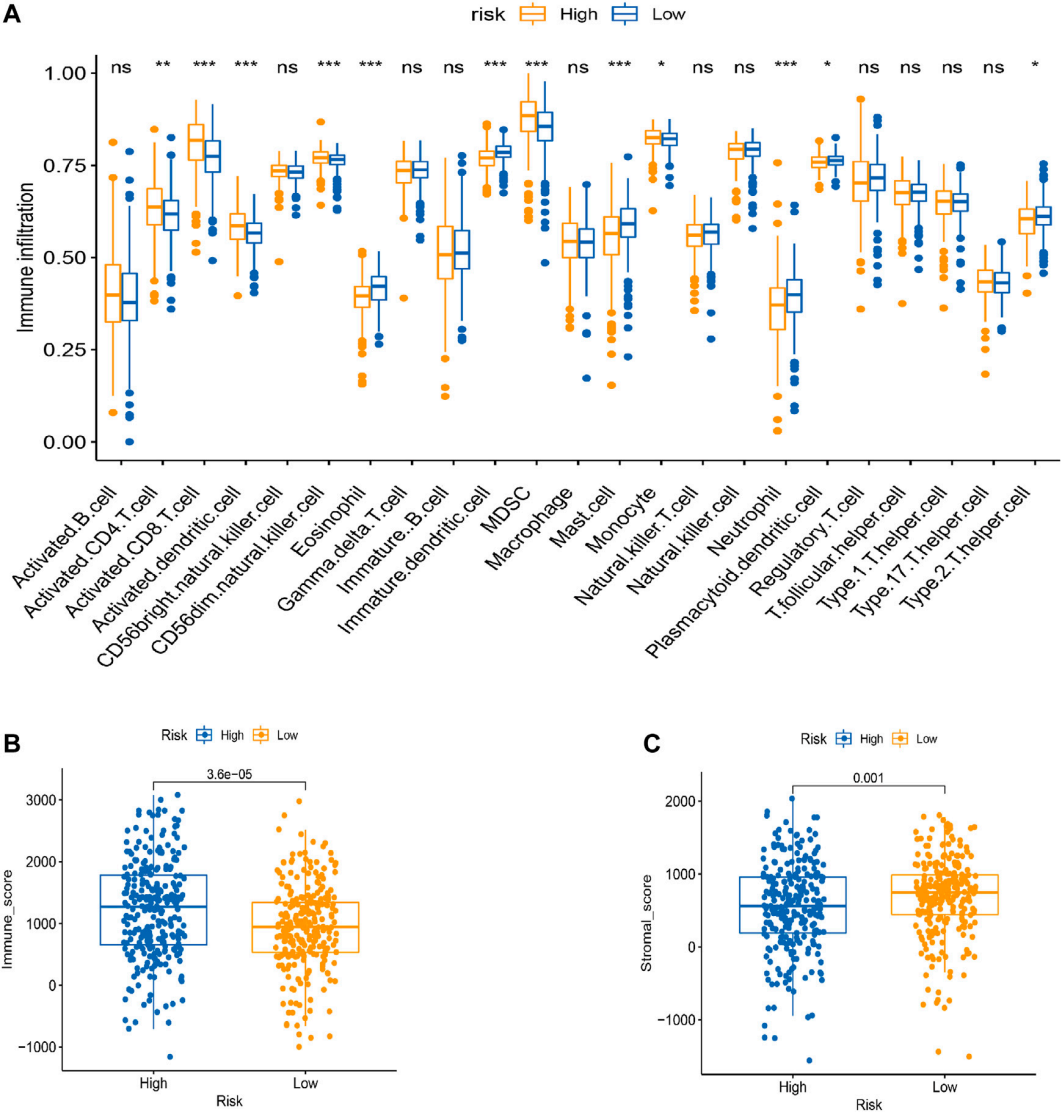
TME cell infiltration characteristics in distinct modification patterns. **(A)** Box plot for the TME cells in distinct risk groups derived from KIRC patients based on the ssGSEA. The asterisks represented the statistical *p* value (*p < 0.05; **p < 0.01; ***p < 0.001). **(B–C)** Immune and stromal scores within the low- and high-risk groups. **(C)** Effect of LRPPRC expression level on the expression of different immunomodulators. *p < 0.05, **p < 0.01, and ***p < 0.001.

Tumor infiltration is crucial for tumor development. Therefore, we performed a ssGSEA analysis to evaluate correlation patterns between immune infiltrating cells and the risk score (Figure 7A). There was significant infiltration of eosinophils, immature dendritic cells, mast cells, and neutrophils in the low-risk group and abundant infiltration of the activated cluster of differentiation (CD) 4 +, CD8^+^ T cells, activated dendritic cells, CD56 dim natural killer (NK) cells, and myeloid-derived suppressor cells (MDSCs) in the high-risk group.

Next, we used the estimation of stromal and immune cells in malignant tumor tissues using the expression data (ESTIMATE) algorithm to identify associations between the risk groups and the immune and stromal scores. The immune score was higher in the high-risk group than in the low-risk group, and the stromal score was higher in the low-risk group than in the high-risk group (Figures 7B,C).

**FIGURE 8:**
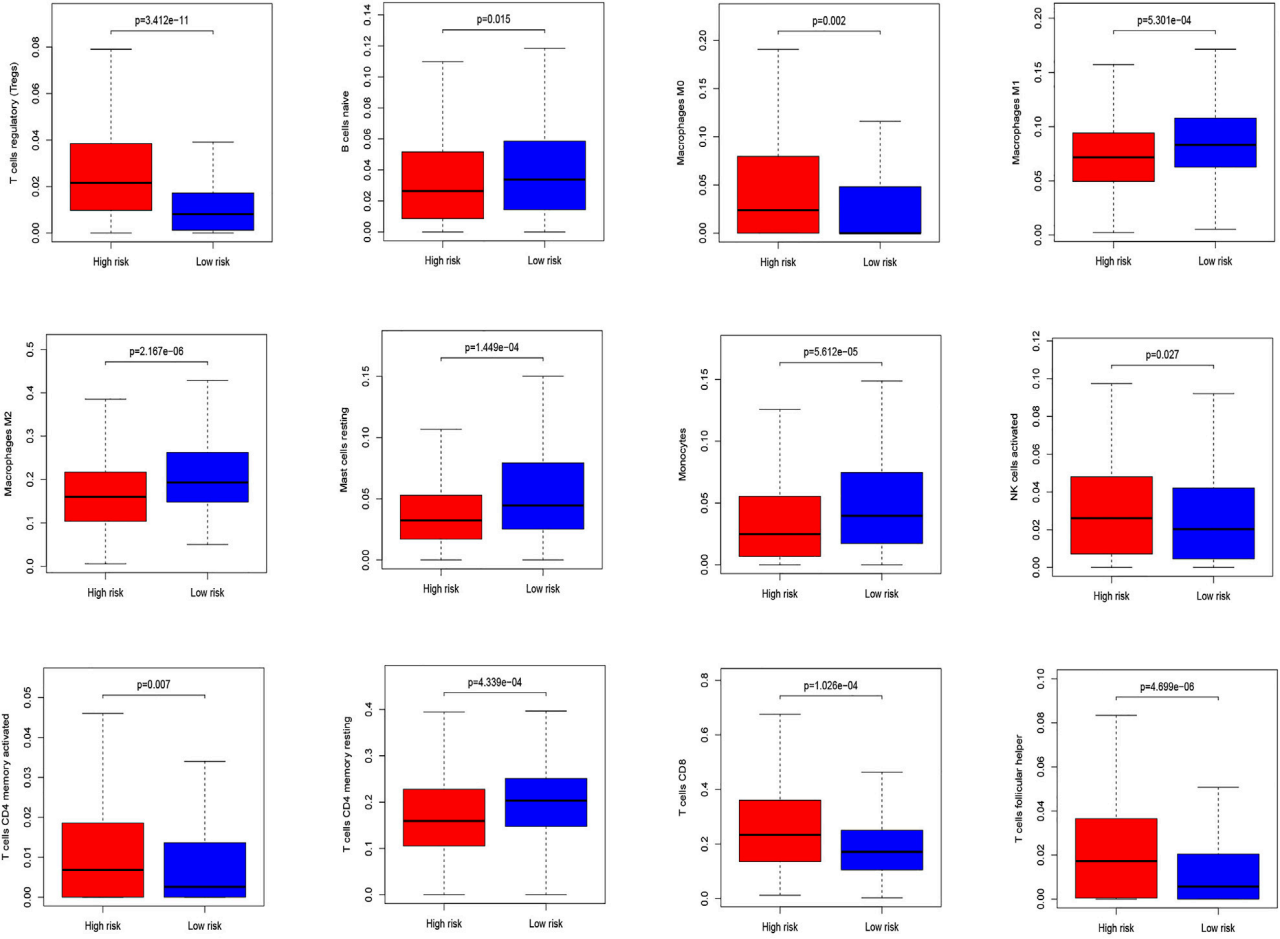
Differential analysis of immune cells in two low-risk groups.

**FIGURE 9:**
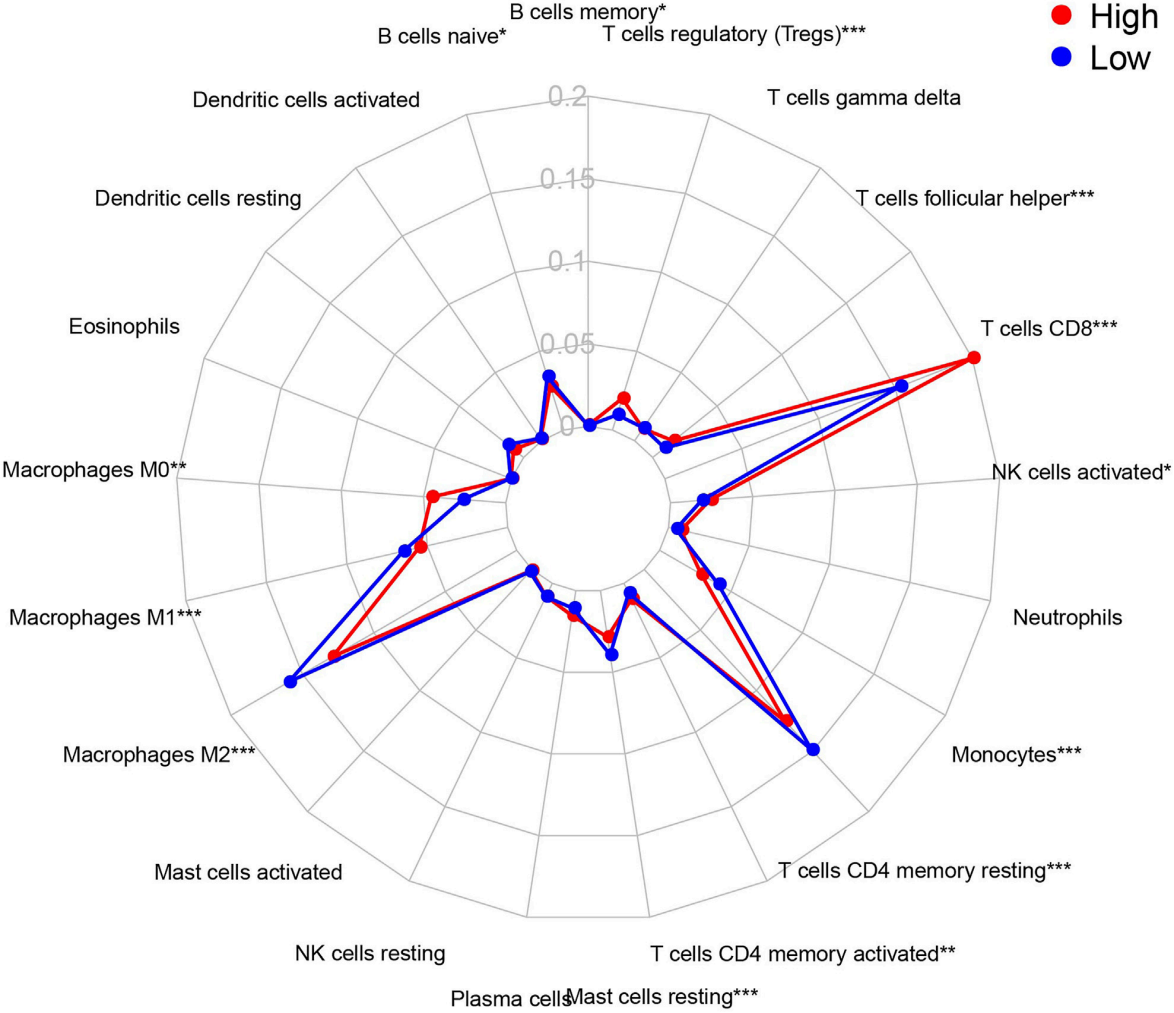
Summary of the 21 immune cells’ abundance for different risk groups of training cohort.

The CIBERSORT algorithm identified the types of immune cells in ccRCC (Figure 8), and we found significant compositional differences between the high- and low-risk groups. M0 macrophages, CD4 memory-activated T cells, CD8 T cells, follicular helper T cells, activated NK cells, and regulatory T cells (Tregs) were significantly enriched in both groups. CD4 resting memory T cells, resting mast cells, M2 and M1 macrophages, monocytes, and naïve B cells were more abundant in the low-risk group than in the high-risk group. Memory B cells did not differ between the groups (Figure 9). These results emphasize that immune cell types vary between high- and low-risk groups. Therefore, exploring immune cell infiltration in ccRCC may help elucidate the mechanisms and improve prognosis predictions.

### Immune Checkpoints Related to the Regulatory Factors

**FIGURE 10:**
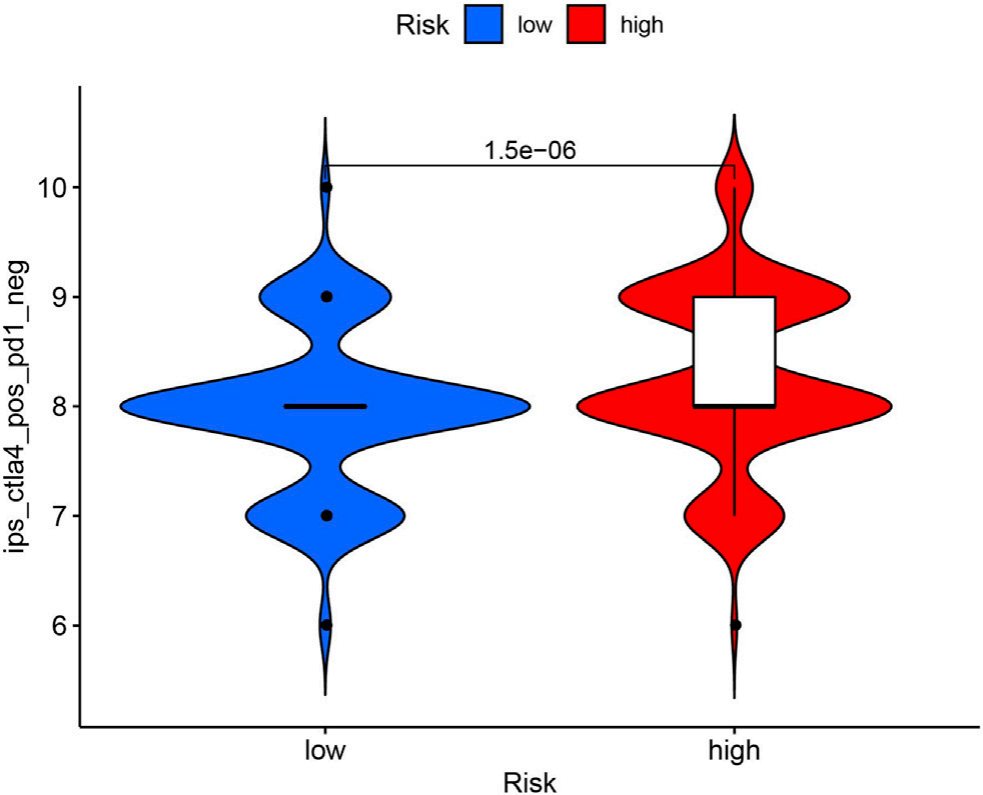
Low-risk group was more likely to respond to anti-CTLA4 immunotherapy. ICPs: immune checkpoints; ICBs: immune checkpoint inhibitors.

Immunotherapy is an emerging ccRCC therapy, and the current first-line treatment involves immune checkpoint inhibitors. Cytotoxic T-lymphocyte-associated protein 4 (CTLA-4) and programmed cell death protein 1 (i.e., PD-1) are the two most important immune checkpoint molecules (ICMs) regarding improved OS (Dunn et al., 2002; Ladányi, 2015; Davis et al., 2019). Other immune checkpoints, such as T-cell immunoglobulin and mucin domain–containing protein 3 (i.e., TIM-3), lymphocyte-activation gene 3 (i.e., LAG-3), and T-cell immunoreceptor with Ig and ITIM domains (i.e., TIGIT), suppress the antitumor immune response (Anderson et al., 2016). Therefore, clarifying the expression correlations of ICMs among risk groups may improve the clinical application of immune checkpoint inhibitors in KIRC. In our study, the immunotherapy score was higher in the high-risk group than in the low-risk group, suggesting that the high-risk group may respond to anti-CTLA-4 treatment (*p* < 0.001; Figure 10).

### *NSUN5* and *HNRNPA2B1* Expression in Kidney Renal Clear Cell Carcinoma

**FIGURE 11:**
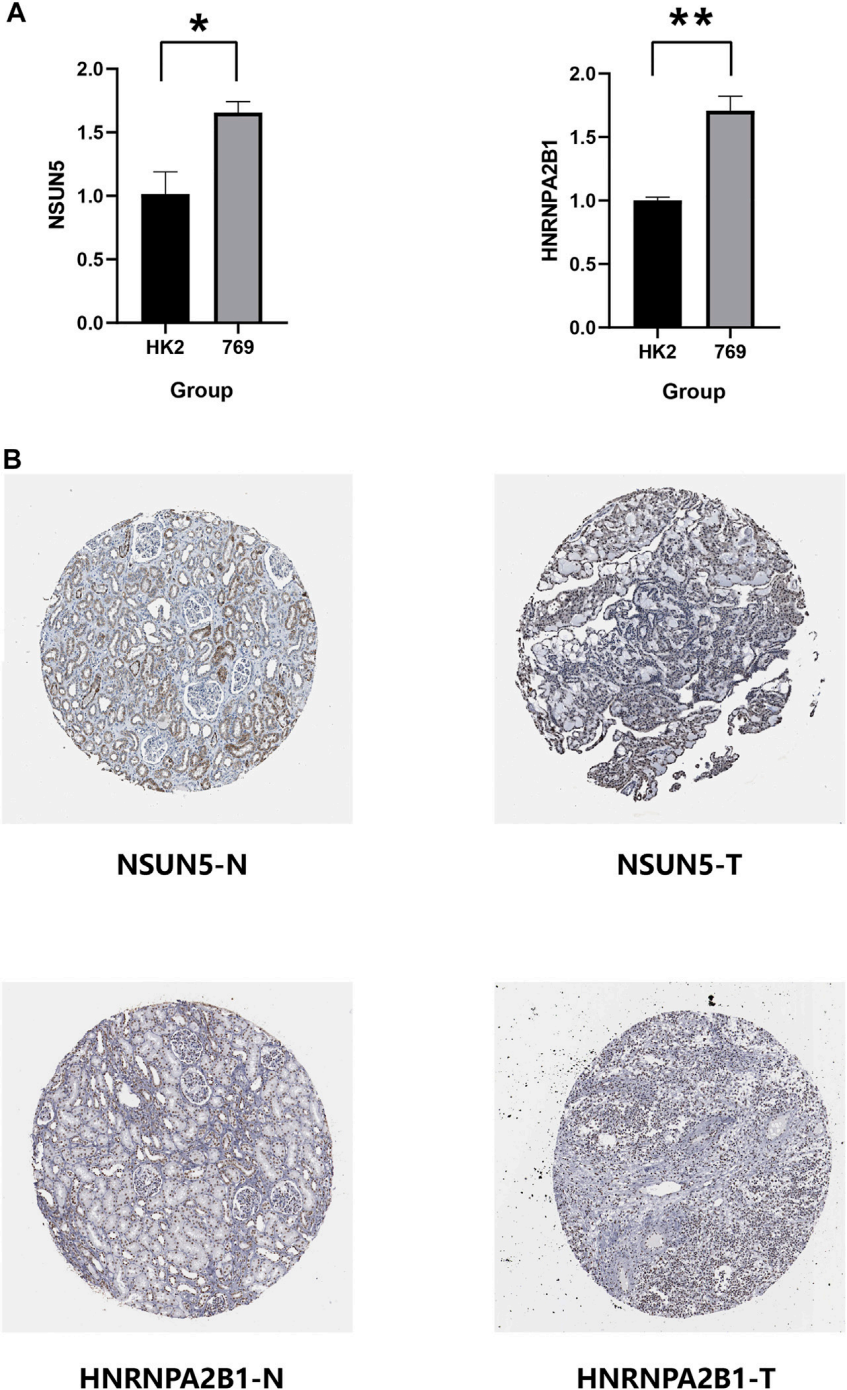
Expression level of NSUN5 and HNRNPA2B1 in renal clear cell carcinoma (KIRC)**. (A)** The expression of NSUN5 and HNRNPA2B1 in ccRCC cell lines (HK2,769) was detected by the qRT-PCR assay. *p < 0.05, **p < 0.01, ***p < 0.001, and ****p < 0.0001. **(B)** Protein expression of NSUN5 and HNRNPA2B1 in KIRC. KIRC, kidney renal clear cell carcinoma.

We verified the expression of *NSUN5*, *ZC3H13*, *METL14*, *NOP2*, and *HNRNPA2B1* in ccRCC using the HK2 (epithelial; control) and 769-P (ccRCC) cell lines. *METL14* is under-expressed in ccRCC tissues (Wang et al., 2021), and *NOP2* expression is higher in tumor tissues than in normal tissues (Li et al., 2021). Furthermore, *ZC3H13* expression is low in tumor tissues (Guo et al., 2021). Based on this, we compared the NSUN5 and HNRNPA2B1 protein levels of ccRCC and normal kidney tissues to determine the *NSUN5* and *HNRNPA2B1* expression status. *NSUN5* and *HNRNPA2B1* expressions were higher in tumor tissues than in normal tissues (Figure 11A), consistent with our other findings. We also compared NSUN5 and HNRNPA2B1 protein levels between RCC and normal kidney tissues, finding more NSUN5 and HNRNPA2B1 staining in RCC tissues than in normal kidney tissues (Figure 11B).

## Discussion

ccRCC is the third most common renal cancer, accounting for 3% of all adult tumors, and the most common sporadic RCC subtype (Padala and Kallam, 2022). Studies have demonstrated that inhibiting m6A and m5C regulatory factors may have therapeutic benefits for tumors, providing a new direction for tumor treatment. For example, R-2-hydroxyglutarate (R-2HG) sensitivity of leukemia increases with increasing the m6A levels (Su et al., 2018). Furthermore, aurora kinase B (i.e., AURKB) regulates NSUN2 at the protein level, phosphorylated by Ser-139 (Sakita-Suto et al., 2007). To date, the relevant ccRCC markers are insufficient for clinical diagnosis and prognosis because ccRCC is often regulated by multiple genes. Thus, a single prognostic factor cannot accurately predict the clinical prognosis. This study explored the key regulators in ccRCC to provide new avenues for cancer treatment by identifying relevant regulatory factors and developing a new gene signature for more accurate prognosis predictions.

Our main goal was to clarify the relationship between m6A and m5C regulatory factors and determine how these combined factors affect KIRC prognosis and immune infiltrating cells. We used data from the TCGA database, identifying 35 relevant m6A and m5C regulatory factors, most of which were abnormally expressed in KIRC. Using these factors, we constructed a new prognostic model for more accurate prediction of OS in patients with KIRC. We randomly divided the TCGA dataset into two groups (training and validation) to verify the effectiveness of the risk model and introduced clinicopathological factors to improve the model’s reliability.

Immune infiltration analysis found more M0 macrophages, CD4 memory-activated T cells, CD8 T cells, follicular helper T cells, activated NK cells, and Tregs in the high-risk group. The low-risk group had more resting CD4 memory T cells, resting mast cells, M1 and M2 macrophages, monocytes, and naïve B cells. Increased eosinophils may be related to a good prognosis, similar to gastric cancer (Iwasaki et al., 1986). However, it could also indicate a poor prognosis, similar to bladder cancer (Popov et al., 2018). Increased number of mast cells are associated with a poor prognosis in lung, colorectal, gastric, and cervical cancers, and melanoma, but it is associated with a good prognosis in breast and prostate cancers (Rajput et al., 2008; Fleischmann et al., 2009; Kormelink et al., 2009). In addition, CD4^+^ T cells promote renal cancer cell proliferation by regulating YBX1 (Wang et al., 2018), and MDSCs accumulate in various tumors, promoting vascular survival and improving tumor immunity (De Cicco et al., 2020). Increased follicular helper T cells and Treg have been shown to promote cancer progression, which relates to a poor prognosis (Finotello and Trajanoski, 2017; Long et al., 2019). The role of mast and dendritic cells and their relationship with renal angiogenesis in KIRC remains unclear (Tamma et al., 2019).

Several studies have suggested that M2 and a small subset of M1 macrophages cannot phagocytose tumor cells and help tumor cells escape death and spread to other tissues and organs. These cells are called tumor-associated macrophages (TAMs) (Zhou et al., 2020), and they promote cancer progression and metastasis in human renal cell carcinoma, stimulating tumor inflammation (Hutterer et al., 2013). TAMs have also been shown to promote tumor metastasis, occurrence, and vascular lymphangiogenesis. During the initial stages of tumor development, macrophages either directly promote antitumor responses by killing tumor cells or indirectly recruit and activate other immune cells (Lopez-Yrigoyen et al., 2021). In our study, there were more M0 macrophages in the high-risk group, consistent with other clinical reports (Yi et al., 2021). Previous studies have shown that low-risk groups have more M1 and M2 macrophages and fewer M0 macrophages than high-risk groups. These descriptions are also consistent with our study’s results. Furthermore, the prognostic outcome of the low-risk group is consistent with the previous survival advantage. The specific role of NK cells remains controversial and largely depends on the cancer type (Hinshaw and Shevde, 2019).

Finally, we used the CIBERSORT and ESTIMATE algorithms to generate immune and stromal scores per risk group. The immune score was higher in the high-risk group than in the low-risk group, and the stromal score was higher in the low-risk group than in the high-risk group. Furthermore, the high-risk group was sensitive to anti-CTLA-4. Liu et al. reported that CTLA-4 as an oncogene accelerates ccRCC development with high prognostic value (Liu et al., 2020).

However, our research has limitations. First, we only have internal verification and lack external platform verification. Therefore, the predictive power of the risk model may be limited. Also, additional basic experimental research is needed to determine the detailed mechanisms of these five regulators.

## Conclusion

Our study evaluated the combined role of m6A and m5C regulatory factors in regulating the KIRC TME. First, we constructed a new gene signature with five relevant regulatory factors. Then, using this signature, we created a novel prognostic model to stratify KIRC and normal tissue samples based on risk. Finally, we explored the link between the new gene signature and immune infiltration and obtained new potential immune checkpoints. These tools may help clinicians make more personalized and accurate prognosis predictions.

## Data Availability

The original contributions presented in the study are included in the article/Supplementary Material, further inquiries can be directed to the corresponding author.

## Supplementary Material

Supplementary Table S1 Different expression of 35 RNA methylation regulators.

Supplementary Table S2 Multivariate Cox regression on the 24 selected regulatory factors.

Supplementary Table S3 Five prognostic factors.
